# Complete Nucleotide Sequence of Canine Distemper Virus CDV-PS, Isolated from Dogs in China

**DOI:** 10.1128/genomeA.00232-13

**Published:** 2013-05-16

**Authors:** Li Yi, Hongli Xu, Jianke Wang, Yuening Cheng, Hailing Zhang, Xijun Yan, Shipeng Cheng

**Affiliations:** Institute of Special Wild Economic Animal and Plant Science, Chinese Academy of Agricultural Sciences, Changchun, Jilin Province, P.R. China

## Abstract

A new strain of canine distemper virus, CDV-PS, has been isolated from dogs in China, and its complete genome has been sequenced and analyzed. The phylogenetic analysis suggests that CDV-PS belongs to the Asia-1 cluster and has low identity to the vaccine strain.

## GENOME ANNOUNCEMENT

Canine distemper (CD) is the most lethal disease of dogs, with signs of generalized infection, including respiratory disease, central nervous system disturbance, footpad hyperkeratosis, or a combination of these symptoms. Canine distemper virus (CDV), the causative agent of canine distemper, is a member of the genus *Morbillivirus*, family *Paramyxoviridae* (1). CDV has long been recognized to cause potentially lethal disease among several species of the carnivores, including dogs, lions, minks, foxes, and raccoon dogs (*Nyctereutes procyonoides*) (2). It has one serotype, and seven different genotypes have been described (Asia-1, Asia-2, Europe, European wildlife, Arctic-like, America-1, and America-2). One case of suspicion of CDV infection was identified initially by the animal owner on the basis of the clinical signs (serous nasal discharge, hemorrhagic enteritis, myoclonus, and fever). Tissue samples were analyzed with a commercial CDV kit (ABD, China) and reverse transcription (RT)-PCR by using a primer pair (forward, AACAAGGCTAGGGTTCAGAC; reverse, TTGTTGACTGATGCAAGACTG) that amplified a 555-bp-long fragment of the N gene, which indicated that the infecting agent was CDV. In addition, the CDV-PS isolate was also identified as CDV by a neutralizing test and an immunofluorescence assay (IFA). The viral RNA was extracted from tissue samples using Trizol reagent (Invitrogen) according to the manufacturer’s instructions. The first-strand cDNA was synthesized from viral RNA using a random primer (Invitrogen). Ten pairs of primers were designed from the published sequences of CDV strains HLJ1-06 (GenBank accession no. JX681125) and Onderstepoort (GenBank accession no. AF305419) to amplify most of the CDV-PS strain genome. The sequences of genome termini were determined by rapid amplification of cDNA ends (RACE) by using a 3′-full RACE kit and 5′-full RACE kit (TaKaRa). The PCR products were cloned into the pMD18-T vector (TaKaRa) and sequenced using an ABI 3730XL Sanger-based genetic analyzer. Sequences were compiled using the SeqMan program in Lasergene. The genome of the CDV-PS strain is 15,690 nucleotides (nt) and consists of six genes in the order of 3′-N–P-M-F–H-L-5′. The levels of identity between the complete genome sequence of the CDV-PS strain and those of 25 other CDVs ranged from 93.0% (for strain Onderstepoort) to 99% (for strain HLJ1-06). The levels of identity for the H gene ranged from 91.0% (for strain Onderstepoort) to 99.3% (for strain BJ080406; GenBank accession no. FJ848535). The phylogenetic analysis and multiple sequence alignment according to the nucleotide sequence of the H gene revealed that CDV-PS belongs to the Asia-1 cluster, which includes most Chinese strains. Nine potential *N*-linked glycosylation sites were found (amino acids [aa] 19 to 21, 149 to 151, 309 to 311, 391 to 393, 422 to 424, 456 to 458, 584 to 586, 587 to 589, and 603 to 605), four of which were identical to those in the Onderstepoort strain.

The complete sequence of the CDV-PS strain offered a detailed analysis of the genetic variation of CDVs in China that is likely to be a helpful to guide efficient preventive, diagnostic, and control strategies for CD in China.

### Nucleotide sequence accession number.

The genome sequence of the CDV-PS strain has been submitted to GenBank (accession number JN896331).
